# Severe Dengue-Associated Hepatitis in an Adolescent Treated With Oral Acetylcysteine: A Case Report

**DOI:** 10.7759/cureus.108434

**Published:** 2026-05-07

**Authors:** Alan Orlando Morales Reyes, Christian Jair Ramos Gomez, Anette Michelle Avila Silva, Estefania Medina Aguilar, Karla Mitchell Rodríguez García

**Affiliations:** 1 Paediatrics, Instituto de Seguridad y Servicios Sociales de los Trabajadores del Estado (ISSSTE), Tampico, MEX; 2 Medicine, Facultad de Medicina de Tampico Dr. Alberto Romo Caballero, Universidad Autonoma De Tamaulipas (UAT), Tampico, MEX; 3 Medicine, Hospital General B, Instituto de Seguridad y Servicios Sociales de los Trabajadores del Estado (ISSSTE), Tampico, MEX

**Keywords:** acute liver injury, dengue, liver enzymes, n-acetylcysteine, pediatric patient, severe hepatitis

## Abstract

Dengue infection may lead to liver involvement ranging from mild transaminase elevation to severe hepatitis and, rarely, acute liver failure. Management is mainly supportive, and no specific therapy exists. We report the case of a 13-year-old adolescent with dengue with warning signs who developed severe hepatitis (aspartate aminotransferase (AST) 2034 U/L, alanine transaminase (ALT) 532 U/L) and thrombocytopenia. Oral N-acetylcysteine (NAC) was initiated as adjuvant therapy. During hospitalisation, a reduction in aminotransferase levels, clinical improvement, and recovery of platelet count were observed. These changes occurred during the course of illness; however, this temporal association does not establish causality and may reflect the expected recovery phase of dengue infection. This case describes the use of NAC as an adjuvant therapy in severe dengue-associated hepatitis. Further studies are required to evaluate its potential role in this setting.

## Introduction

Dengue infection is a mosquito-borne viral disease that represents a major public health problem in tropical and subtropical regions worldwide, with millions of cases reported annually and a subset progressing to severe disease [1]. Hepatic involvement is a common manifestation of dengue infection and may range from mild elevations of aminotransferases to severe hepatitis and, rarely, acute liver failure. While most cases are self-limited, some patients may develop significant hepatocellular injury associated with complications during the critical phase of the disease.

The pathophysiology of liver injury in dengue is complex and multifactorial, involving direct viral cytopathic effects; immune-mediated damage; hypoxia due to plasma leakage and shock; and oxidative stress [2,3]. These mechanisms may lead to hepatocellular necrosis and marked elevation of aminotransferases. Management of dengue-associated hepatitis remains primarily supportive, as no specific antiviral therapy targeting dengue-related liver injury is currently available [4]. In this context, N-acetylcysteine (NAC), a precursor of glutathione with antioxidant and cytoprotective properties, has been explored as a potential adjuvant therapy in various forms of acute liver injury [5-7].

Although NAC has demonstrated benefit in non-acetaminophen-induced acute liver failure, evidence supporting its use specifically in dengue-associated hepatitis remains limited and is largely derived from case reports and small case series [8-15]. Furthermore, its role outside of established acute liver failure is not well defined. In this context, we present the case of an adolescent with severe dengue-associated hepatitis who received oral NAC as part of adjuvant management. This report aims to describe the clinical course and highlight the temporal association between NAC administration and biochemical improvement, while acknowledging the self-limited nature of dengue infection and the inability to establish causality.

## Case presentation

We report the case of a 13-year-old female adolescent from Mexico who presented on day one of illness with fever (not quantified), headache, myalgia, arthralgia, somnolence, and abdominal pain. Outpatient laboratory testing revealed a positive dengue NS1 antigen, with negative IgM and IgG, consistent with acute dengue infection. On day three, she presented to the emergency department with persistent symptoms and was initially classified as dengue without warning signs (group A). She was discharged with outpatient management and home monitoring. However, within hours, she developed persistent vomiting, diarrhea, gingival bleeding, and worsening abdominal pain, prompting reassessment.

**Figure 1:**
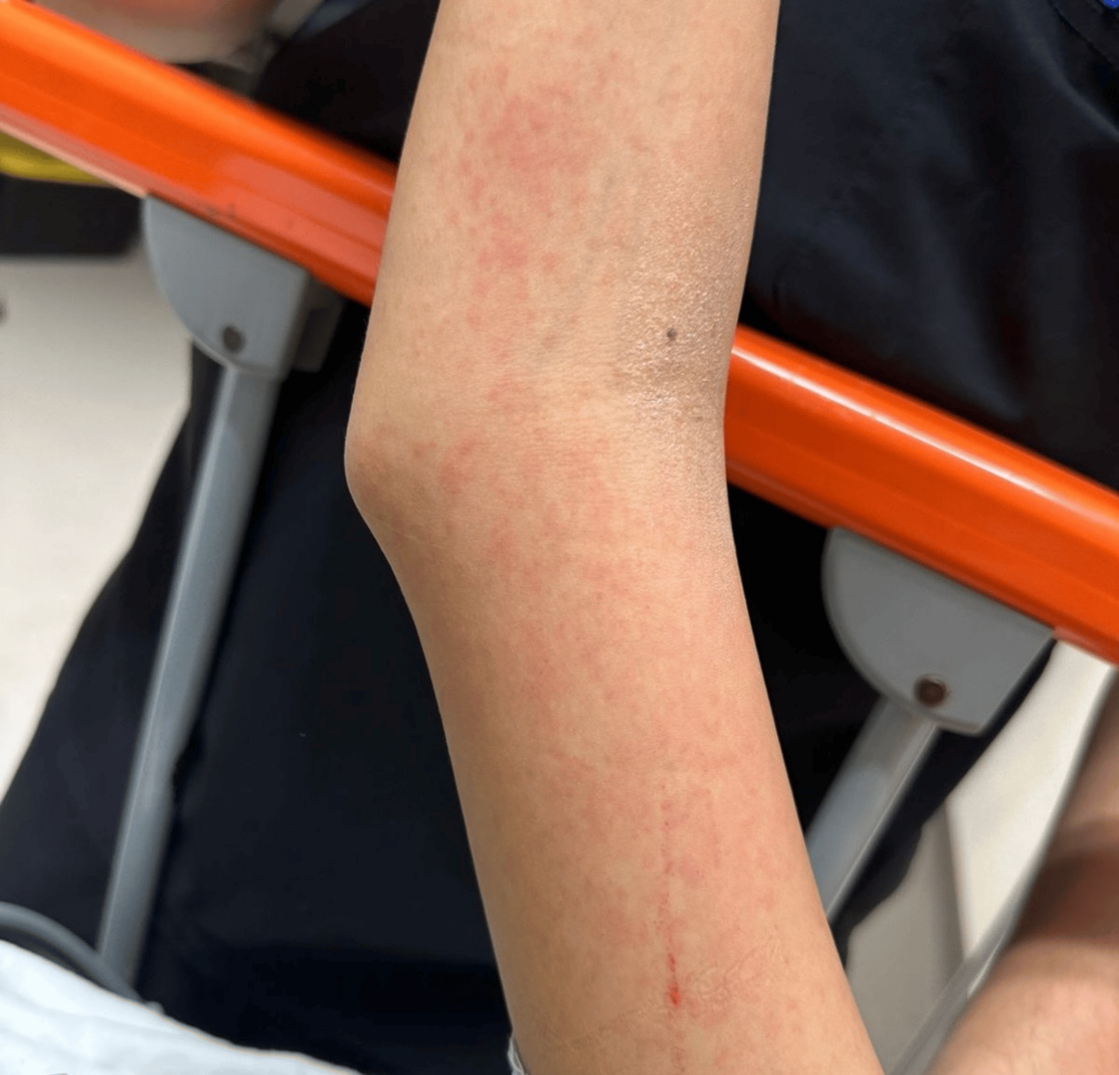
Maculopapular rash involving the upper extremity, characterized by diffuse erythematous lesions with linear excoriation suggestive of dermographism, consistent with cutaneous manifestations observed during dengue infection.

**Figure 2:**
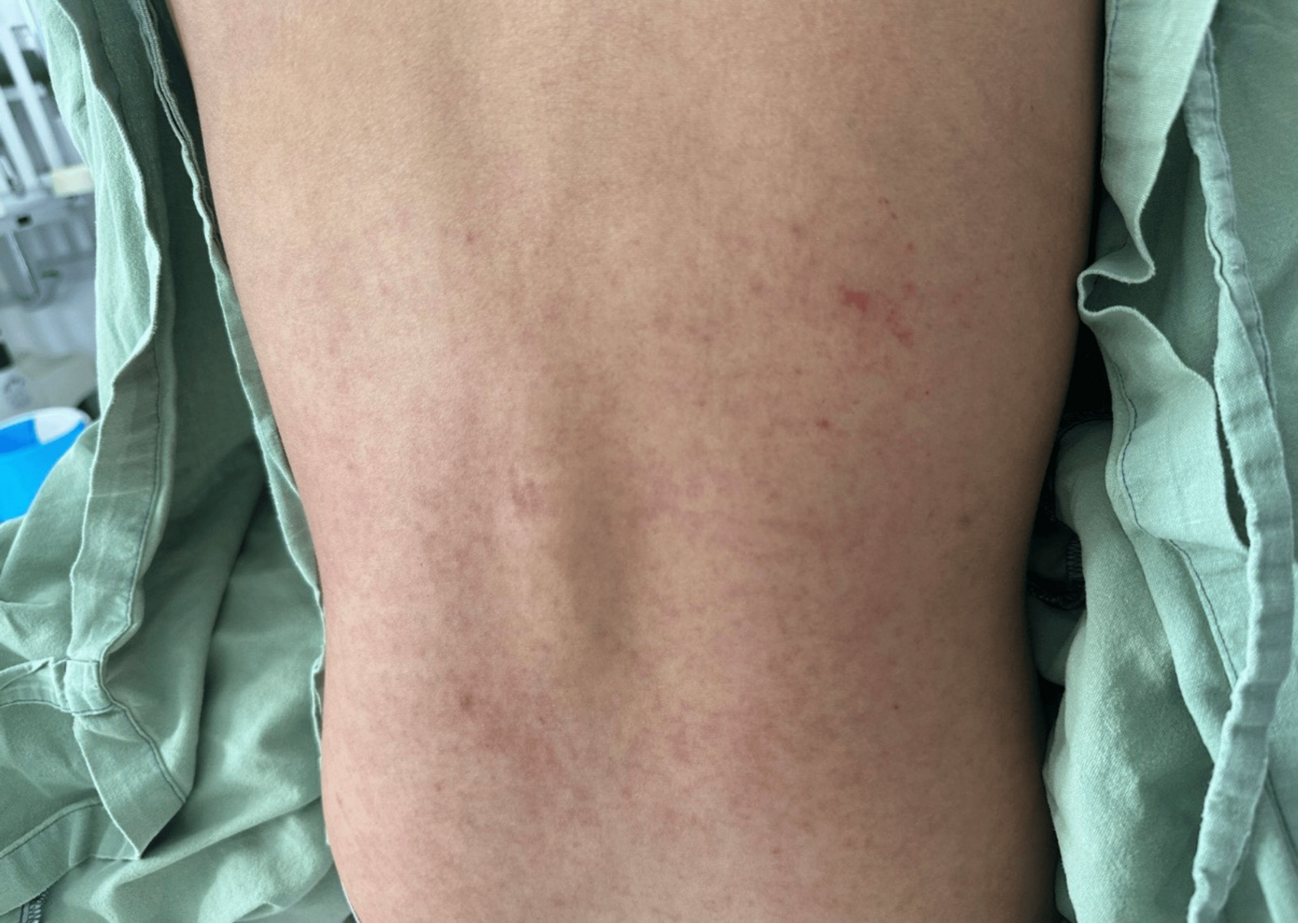
Generalized maculopapular rash on the trunk, characterized by diffuse erythematous macules and papules, consistent with cutaneous manifestations of dengue during the critical phase.

On day five, she was admitted with dengue with warning signs (group B) in the critical phase. Warning signs included persistent vomiting, abdominal pain, mucosal bleeding, and thrombocytopenia. On physical examination, she was conscious and oriented, hemodynamically stable, with mildly dehydrated mucous membranes and diffuse abdominal tenderness. Mild scleral icterus was noted. Cutaneous findings included a maculopapular rash on the upper extremities with linear excoriations suggestive of dermographism (Figure 1), as well as a generalized rash on the trunk (Figure 2). No neurological impairment or respiratory distress was observed.

During hospitalization, progressive leukopenia and thrombocytopenia were documented, with a nadir platelet count of 40-45 ×10³/µL. Hematocrit values were not consistently available. Marked elevation of liver enzymes was observed, with a peak aspartate aminotransferase (AST) of 2034 U/L and alanine transaminase (ALT) of 532 U/L, consistent with acute dengue-associated hepatitis. Additional parameters relevant for liver failure assessment, including international normalized ratio (INR)/prothrombin time (PT), serum ammonia, lactate, albumin, and creatine phosphokinase, were not available. Laboratory trends are summarized in Table 1.

**Table 1:** Laboratory findings during the course of illness The table demonstrates progressive leukopenia, thrombocytopenia, and marked elevation of aminotransferases during the critical phase of dengue, followed by hematological and biochemical improvement during the recovery phase. Reference ranges: Leukocytes 4-10 ×10³/µL; platelets 150-450 ×10³/µL; AST <40 U/L; ALT <40 U/L; total bilirubin <1.2 mg/dL AST: Aspartate aminotransferase, ALT: Alanine transaminase

Day of illness	Leukocytes (×10³/µL)	Hemoglobin (g/dL)	Platelets (×10³/µL)	AST (U/L)	ALT (U/L)	Total bilirubin (mg/dL)	Creatinine (mg/dL)
Day 1	4.6	14.03	223	-	-	-	-
Day 2	4.77	13.4	160	-	-	-	0.8
Day 3	3.13	14.0	113	234	94	0.37	0.5
Day 4	2.49	14.3	66	2034	532	0.72	-
Day 5	-	-	-	1600	537	0.91	-
Day 6	3.61	15.6	40	-	-	-	-
Day 7	7.75	13.8	82	925	409	0.66	0.5

Given the marked elevation of aminotransferases and concern for potential progression of hepatic injury, oral NAC was initiated as adjuvant therapy with a loading dose of 140 mg/kg, followed by 70 mg/kg every four hours. This decision was based on clinical judgment and previous reports of NAC use in acute liver injury; however, no institutional protocol was followed. Supportive management included intravenous fluid therapy, close hemodynamic monitoring, and serial laboratory evaluation.

**Figure 3:**
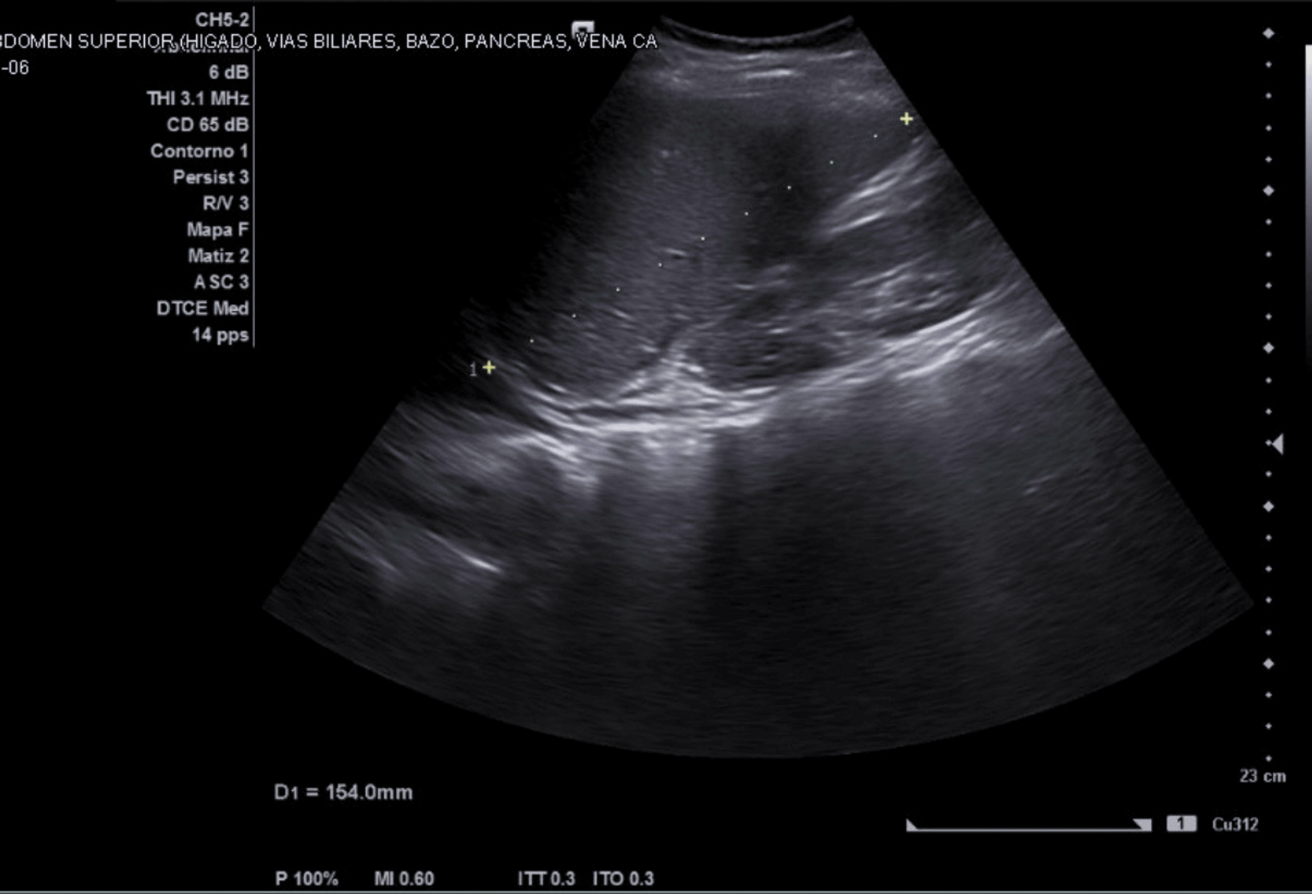
Abdominal ultrasound demonstrating hepatomegaly with increased liver span (154 mm), consistent with hepatic involvement in dengue infection.

**Figure 4:**
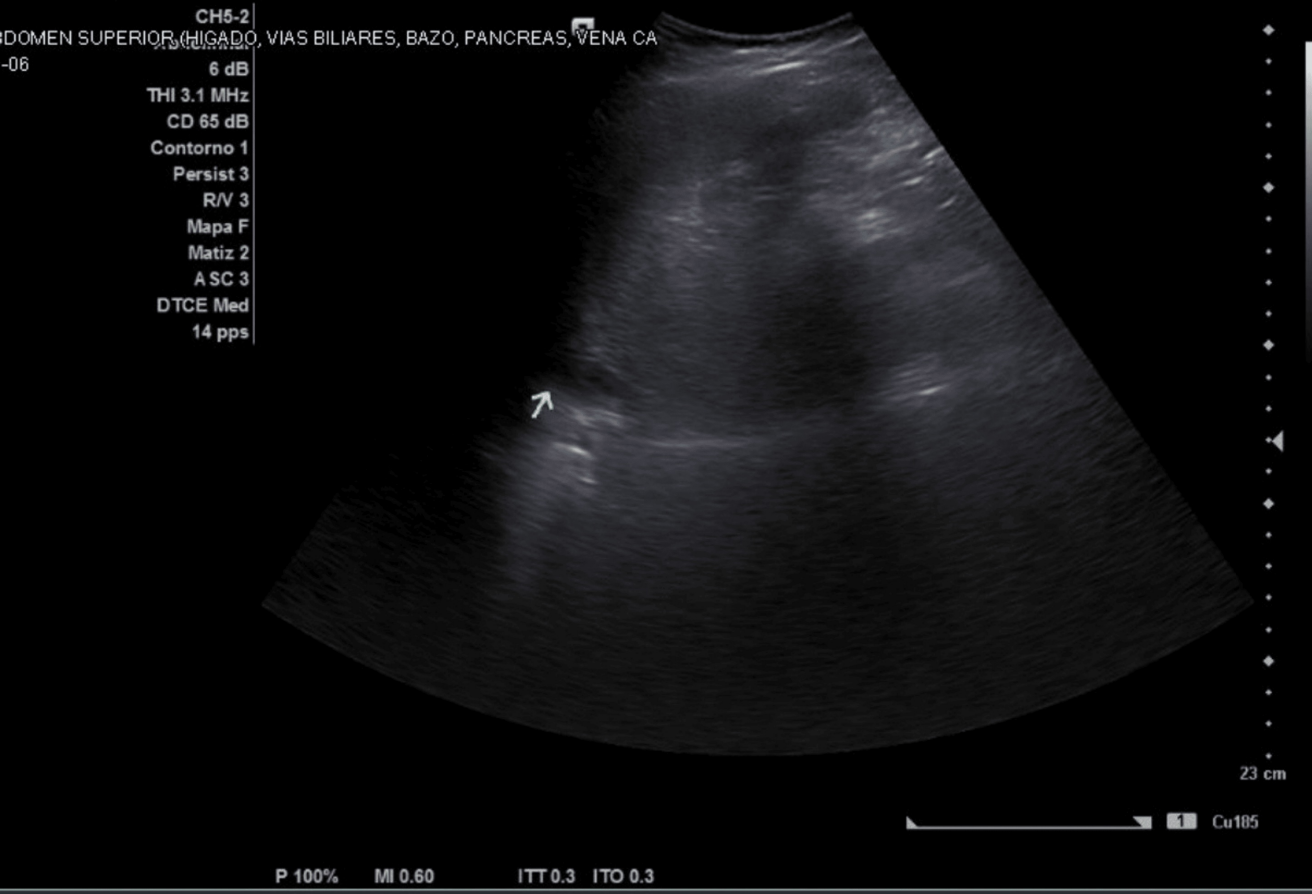
Abdominal ultrasound demonstrating perihepatic free fluid (arrow), consistent with plasma leakage during the critical phase of dengue infection.

Abdominal ultrasound demonstrated hepatomegaly (Figure 3), perihepatic free fluid (Figure 4), and small bilateral pleural effusions, findings consistent with plasma leakage during the critical phase of dengue infection. During hospitalization, a decline in aminotransferase levels, clinical improvement, and recovery of platelet count were observed. The AST decreased from 2034 U/L to 925 U/L and ALT from 532 U/L to 409 U/L. Platelet count also increased to 82 ×10³/µL. These changes occurred after the peak of liver enzyme elevation and during the expected recovery phase of dengue. The evolution of liver enzymes is summarized in Table 2. The patient remained hemodynamically stable, without hemorrhagic or neurological complications, and showed gradual clinical improvement. She was discharged on day eight of illness in the convalescent phase, with outpatient follow-up.

**Table 2:** Evolution of liver enzymes after initiation of NAC This table summarizes the changes in liver enzyme levels during hospitalization. A reduction in aminotransferase levels was observed (AST decreased by 54% and ALT by 23%); however, this temporal association does not establish causality and may reflect the expected recovery phase of dengue infection. NAC: N-acetylcysteine, AST: Aspartate aminotransferase, ALT: Alanine transaminase

Parameter	Peak value	After NAC	Percentage change
AST	2034 U/L	925 U/L	-54%
ALT	532 U/L	409 U/L	-23%

## Discussion

Dengue is one of the most common vector-borne viral infections worldwide and represents a major public health problem in tropical and subtropical regions. Although most cases follow a self-limited mild course, a proportion of patients may develop severe complications, including significant hepatic involvement. Liver involvement in dengue is frequent and may range from mild elevations of transaminases to severe acute hepatitis and, rarely, fulminant liver failure [1,2].

The pathophysiological mechanisms of liver injury in dengue are multifactorial and include direct viral cytopathic effects, immune-mediated injury, hepatic hypoxia due to plasma leakage, microcirculatory disturbances, and oxidative stress. These processes can result in hepatocellular injury and marked elevation of aminotransferases, particularly during the critical phase of the disease [1,3]. In the present case, the patient developed marked elevation of liver enzymes, with a peak AST of 2034 U/L and ALT of 532 U/L, consistent with acute dengue-associated hepatitis. The predominance of AST over ALT has been previously described and may reflect both hepatic and extrahepatic (muscle-related) injury [1,4].

N-acetylcysteine has been explored as an adjuvant therapy in non-acetaminophen-induced acute liver injury due to its antioxidant properties, its role in glutathione replenishment, and its potential to improve hepatic perfusion [5,6]. Some studies in acute liver failure have suggested a possible benefit in selected populations, particularly in early stages of disease [5-7]. However, in the specific context of dengue-associated hepatitis, the available evidence is limited and largely based on case reports and small case series. Although some reports describe clinical and biochemical improvement following NAC administration, these findings are difficult to interpret due to the self-limited nature of dengue and the absence of controlled comparisons [12-15].

In this case, a reduction in aminotransferase levels was observed during the clinical course. However, this temporal association does not establish causality, as the improvement occurred during the expected recovery phase of dengue (days six to seven), when spontaneous resolution of hepatic injury and platelet recovery typically occur. Therefore, the observed biochemical improvement should be interpreted with caution and may reflect the natural course of the disease rather than a treatment effect. This is particularly relevant given the absence of markers of acute liver failure, such as coagulopathy or encephalopathy, and the lack of complete laboratory data to assess severity. Despite these limitations, NAC remains an attractive candidate for further investigation given its favorable safety profile, low cost, and biological plausibility. Nevertheless, current evidence is insufficient to support its routine use in dengue-associated hepatitis outside of research settings or selected clinical scenarios.

The main limitations of this report include its single-case design, the absence of a control group, incomplete laboratory data (including INR, ammonia, lactate, and creatine phosphokinase), and the inability to exclude all alternative causes of liver injury. These factors limit the interpretation of the findings and preclude any causal inference. Further prospective studies and controlled trials are required to determine whether NAC provides a true clinical benefit in patients with dengue-associated hepatic involvement, particularly in pediatric populations.

## Conclusions

Dengue-associated hepatitis is a potentially serious complication that may occur during the critical phase of the disease and, in rare cases, progress to acute liver failure. In this case, an adolescent with dengue and warning signs developed marked elevation of liver enzymes consistent with acute dengue-associated hepatitis. A reduction in aminotransferase levels was observed following the initiation of oral NAC; however, this temporal association does not establish causality and may reflect the expected natural recovery phase of dengue infection. N-acetylcysteine has been proposed as an adjuvant therapy in non-acetaminophen-related acute liver injury due to its antioxidant and hepatoprotective properties. Nevertheless, current evidence on dengue-associated hepatitis remains limited and is primarily derived from observational reports.

This case highlights the clinical course of dengue-associated hepatic involvement and the use of NAC in this context, but no conclusions regarding efficacy can be drawn. Further prospective and controlled studies are required to determine whether NAC provides a true clinical benefit in this setting.
